# Comparison between the outcomes of manipulation under anaesthesia versus intra-articular steroid injection in idiopathic adhesive capsulitis

**DOI:** 10.6026/973206300214409

**Published:** 2025-12-15

**Authors:** Raju Manda, Ashoke Kumar Chanda, Sudipta Dasgupta, Gourav Naskar, Kousik Biswas

**Affiliations:** 1 Department of Orthopaedics, Calcutta National Medical College & Hospital, Kolkata, West Bengal 700014, India

**Keywords:** Steroid, shoulder, symptoms, manipulation under anaesthesia (MUA), frozen shoulder

## Abstract

One of the most common causes of shoulder pain and restricted motion is frozen shoulder, or adhesive capsulitis, characterized by a thickened, contracted joint capsule with chronic inflammatory changes. Therefore, it is of interest to compare the outcomes of manipulation under anaesthesia (MUA) and intra-articular steroid injection in idiopathic adhesive capsulitis. A total of 60 patients were prospectively observed and treated at the Department of Orthopaedics, Calcutta National Medical College and Hospital, from April 2021 to September 2022. The mean time for resolution of symptoms was 11.30±0.41 weeks in the MUA group and 10.90±0.67 weeks in the steroid group, with a statistically significant difference (p=0.0073). Thus, MUA was found to be particularly effective for rapid functional restoration, providing quicker improvement in shoulder mobility and early pain relief.

## Background:

Frozen shoulder (FS), or adhesive capsulitis, is a condition characterized by joint stiffness, reduced motion and persistent pain at the end range of movement, even though pain at rest may decrease over time [1]. The pathological hallmark of FS involves a thickened and contracted joint capsule, chronic synovial inflammation and a marked reduction in synovial fluid [2]. Although the exact etiology remains unclear, several contributing factors, including inflammation, fibrosis and capsular contracture, are believed to play a role in the development of the condition [3]. Conservative treatments such as physiotherapy, analgesics and corticosteroid injections are commonly employed and have shown effectiveness in reducing pain and improving function in many cases [4]. Minimally invasive procedures, such as manipulation under anaesthesia (MUA), aim to break capsular adhesions mechanically, offering rapid improvements in range of motion and symptom relief [5]. However, there is limited high-quality evidence directly comparing the efficacy of these interventions [6]. Among non-surgical options, intra-articular corticosteroid injections (ISI) have also demonstrated positive outcomes in clinical trials, providing significant short-term pain relief and functional improvement [7]. Frozen shoulder affects approximately 2% to 5% of the general population, with a higher prevalence in individuals aged 40-60 years and in women [8]. This condition is commonly associated with significant impairments in daily activities, work function and sleep quality due to pain and limited shoulder mobility [9]. Although FS is often described as self-limiting and may resolve spontaneously over 1-3 years, some cases progress to chronic stiffness and persistent functional deficits [10]. The etiology of primary (idiopathic) FS remains unknown, but it is widely accepted that capsular fibrosis and inflammation are central to its pathophysiology, explaining the characteristic pain and stiffness [3]. Treatment modalities span a spectrum from conservative (e.g., physiotherapy, NSAIDs, acupuncture and corticosteroid injections) to more aggressive interventions (e.g., MUA or arthroscopic capsular release) [11]. However, the relative effectiveness of these approaches remains a topic of on-going research and debate. Thus, there is a clinical need and growing interest in comparing the outcomes of manipulation under anaesthesia and intra-articular steroid injections in the management of idiopathic adhesive capsulitis, to guide optimal treatment strategies [12]. Therefore, it is of interest to compare between the outcomes of manipulation under anaesthesia versus intra-articular steroid injection in idiopathic adhesive capsulitis.

## Materials and Methods:

## Study design:

Prospective, Observational and Comparative Study.

## Place of Study:

Department of Orthopaedics, Calcutta National Medical College and Hospital.

## Period of Study:

April 2021- September 2022.

## Study population:

Patients visiting Orthopaedics Outdoor Patient Department (OPD) in Calcutta National Medical College and Hospital with frozen shoulder aged 30-70 years old.

## Sample size:

100 Adhesive Capsulitis Patients.

## Inclusion criteria:

[1] Age: 30-70 years.

[2] Both sexes.

[3] Spontaneous onset of a painful stiff shoulder.

[4] Patient who got exhausted after conservative treatment such as analgesics and physiotherapy.

[5] Symptoms present for at least 6 weeks.

[6] Normal x-rays on antero-posterior and axillary lateral radiographs of the gleno-humeral joint.

## Exclusion criteria:

[1] Radiographic pathological findings or gleno-humeral osteoarthritis on X-ray.

[2] Significant cervical spine disease.

[3] History of significant trauma to the shoulder.

[4] Local corticosteroid injection or any physiotherapy intervention to the affected shoulder within the last 3 months.

[5] Bilateral frozen shoulder due to possible underlying systemic cause.

[6] Inflammatory joint disease affecting the shoulder.

[7] Patient attending with pain and stiffness in emergency department are excluded.

## Procedure:

Patients attending in Outdoor Patient Department (OPD) with pain and stiffness in shoulder joint were admitted and simple randomization was done into two groups by lottery system. 54

[1] Group 1 (n=30) comprised intra-articular injection patients.

[2] Group 2 (n=30) included patients who received manipulation under anaesthesia.

The patients were prohibited from using any other adjuvant therapy apart from analgesics like non-steroidal anti-inflammatory drugs during the study period.

## In Group 1:

The patients who fulfilled the study inclusion and exclusion criteria, an intra- articular steroid injection were applied in accordance with the method disclosed. Before application of the intra-articular injection, the patient's affected shoulder area was swabbed three times with 5% povidone iodine solution. Intra-articular injection given in a posterior approach containing a mixture of 2ml of 2% lignocaine Hydrochloride (xylocaine) and 2ml (80 mg) methylprednisolone acetate via a 10ml needle. All patients were injected once. Active and passive range of movement (AROM and PROM) assessments was performed before and after injection and at all subsequent visits.

## In Group 2:

MUA (manipulation under anaesthesia) (n= 30) was done on elective operation list under general anaesthesia using a short lever arm and fixed scapula and in supine position. During the procedure, a typical rasping noise occurred during manipulation which confirms the clinical diagnosis of frozen shoulders; the affected extremity was lifted and pushed the upper arm in flexion and abduction. After that shoulder was stretched into flexion, the elbow was flexed to a right angle and the upper arm was gently rotated into internal and external rotation. Audible and palpable release of adhesions is a good prognostic sign.

## Follow-up:

Patients were assessed after 4weeks. Pain was measured at rest, the patient was asked to identify the worst pain they felt when the shoulder is in rest, using the visual analogue scale (VAS). Scale values were then obtained by measuring the distance marked by the patient from zero to 10cm, 0 being no pain and 10 being the worst pain. The shoulder pain and disability index (SPADI) were handed as a self-administered questionnaire. It is designed by Roach and colleagues to measure the impact of shoulder pathology in terms of pain and disability, for both current status and change over time to produce a total score ranging from 0 (best) to 100 (worst).

## Statistical analysis plan:

A total of 60 patients of frozen shoulder were studied using descriptive statistics. Chi square test was used to determine the association between two variables. P value of less than or equal to 0.05 was taken as significant. The available version of statistical package (IBM SPSS Statistics Version 26) was used. All data was kept confidential and preserved for at least 3 years.

## Statistical analysis:

For statistical analysis data were entered into a Microsoft excel spreadsheet and then analyzed by SPSS (version 27.0; SPSS Inc., Chicago, IL, USA) and Graph Pad Prism version 5. Data had been summarized as mean and standard deviation for numerical variables and count and percentages for categorical variables. Two-sample t-tests for a difference in mean involved independent samples or unpaired samples. Paired t-tests were a form of blocking and had greater power than unpaired tests. A chi-squared test (χ^2^ test) was any statistical hypothesis test wherein the sampling distribution of the test statistic is a chi-squared distribution when the null hypothesis is true. Without other qualification, 'chi-squared test' often is used as short for Pearson's chi-squared test. Unpaired proportions were compared by Chi-square test or Fischer's exact test, as appropriate. Explicit expressions that can be used to carry out various t-tests are given below. In each case, the formula for a test statistic that either exactly follows or closely approximates a t-distribution under the null hypothesis is given. Also, the appropriate degrees of freedom are given in each case. Each of these statistics can be used to carry out either a one-tailed test or a two-tailed test. Once a t value is determined, a p-value can be found using a table of values from Student's t- distribution. If the calculated p-value is below the threshold chosen for statistical significance (usually the 0.10, the 0.05, or 0.01 level), then the null hypothesis is rejected in favor of the alternative hypothesis. P-value ≤ 0.05 was considered for statistically significant.

## Results and analysis:

The mean (mean± standard deviation) Vas Score Pre-Management for patients in MUA was 7.0000 ±.6433. The patients' mean Vas Score before to management (mean ± standard deviation) in Steroid was 7.4000 ±.8137. The distribution of the pre-management mean Vas Score according to the kind of operation showed statistical significance (p=0.0390). The patients' mean Vas score at 4 weeks (mean ± standard deviation) in MUA was 6.4000±.8137. After four weeks of Steroid treatment, the mean Vas score (mean ± standard deviation) for the patients was 6.6000± 1.0372. The mean Vas score distribution after four weeks of management according to the type of surgery was not statistically significant (p=0.4094). The patients' mean Vas score after 8 weeks of management (mean ± standard deviation) was 3.6000 ± 3.2000 in MUA. After 8 weeks of treatment with steroids, the mean Vas score (mean± standard deviation) for the patients was 3.2000±.9965. The distribution of the mean Vas score eight weeks after treatment, broken down by surgical type, did not show statistical significance (p=0.1332). The patients' mean Vas score after 12 weeks of management (mean ± standard deviation) in MUA was 1.2000 ±.7611. The patients' mean Vas score after 12 weeks of Steroid treatment was 1.0000±.6433 (mean± s.d.). The mean Vas score distribution after 12 weeks of management according to the type of surgery was not statistically significant (p=0.2762) (Table 1 - see PDF). The mean Time of Resolution of Symptoms (Wks) (mean ± standard deviation) for patients in MUA was 11.3000± 0.4068.In Steroid, patients' mean Time of Symptom Resolution (Wks) (mean ± s.d.) was 10.9000±.6747. There was a statistically significant distribution of the mean Time of Resolution of Symptoms (Wks) with Type of Surgery (p=0.0073) (Figure 1 - see PDF).

## Discussion:

Our study examined 60 patients to compare the effectiveness of Manipulation under Anaesthesia (MUA) and intra-articular steroid injections in managing adhesive capsulitis of the shoulder. The mean age of patients treated with steroids (56.2 ± 6.7 years) was slightly higher than those receiving MUA (52.6 ± 8.5 years) and 41.7% of patients were over 50 years old, with no significant age-based differences (p=0.1729). Overall, males constituted 58.3% of the study population, though the distribution of frozen shoulder was similar between genders (male: 56.7%, female: 60.0%, p=0.7934). Patient satisfaction was higher in the steroid group (80.0%) compared to the MUA group (56.7%), a difference that was statistically significant (p=0.0015), aligning with previous reports highlighting steroid efficacy in symptom management [13]. The average symptom duration was slightly longer in the steroid group (10.8 ± 2.5 weeks) than in the MUA group (10.5 ± 2.2 weeks). Pre-treatment VAS scores were significantly higher in the steroid group (7.4 ± 0.8) than in the MUA group (7.0 ± 0.6, p=0.0390), though follow-up scores at 4, 8 and 12 weeks showed no significant differences. Similarly, pre-treatment SPADI scores were marginally higher in the MUA group (7.4 ± 0.8) than in the steroid group (7.0 ± 0.6, p=0.5141), with no significant differences at subsequent follow-ups, supporting earlier findings that steroids combined with physiotherapy can improve functional outcomes [14]. Pre-treatment abduction was higher in the MUA group (57.0 ± 8.9°) compared to the steroid group (52.0 ± 6.9°, p=0.0179), while external rotation was higher in the steroid group (29.0 ± 3.8° vs. 29.0 ± 2.0°, p < 0.0001); however, post-treatment differences at 4, 8 and 12 weeks were not significant. These results are consistent with prior studies demonstrating that steroid injections effectively enhance shoulder range of motion over time [15]. Comparative literature suggests that while surgical interventions may yield greater pain reduction, image-guided steroid injections remain an effective, less invasive option when precision and follow-up are ensured [14, 16]. Veeraraghavan *et al.* (2020) [17] found that MUA alone provided quicker functional improvement in the early phase (up to 1 month), but by 3-6 months, outcomes were comparable between MUA and steroid injection, indicating faster but not necessarily superior long-term benefits with MUA. Saeed *et al.* (2022) [18] highlighted that steroid injection was safer, easier, and less costly than MUA, offering slightly better short-term pain relief, though differences between the treatments were not statistically significant. In the present study, patients treated with manipulation under anaesthesia (MUA) and those receiving steroid therapy both demonstrated substantial reductions in mean VAS scores over 12 weeks, with a trend toward faster symptom resolution in the steroid group (mean resolution 10.9 weeks vs. 11.3 weeks; p = 0.0073). These findings echo recent evidence in the literature. For instance, Kayaokay *et al.* [19] (2023) compared MUA alone versus MUA combined with intra-articular corticosteroids, and noted that while pain relief (VAS) was similar by 12 weeks and 6 months, the addition of steroids provided superior early pain control.

## Conclusion:

We show that MUA is suitable for cases requiring rapid functional recovery, as it quickly improves shoulder mobility and offers early pain relief. In contrast, intra-articular steroid injection provides longer-lasting pain control and may be preferable for patients seeking sustained relief with fewer procedural risks. Clinical decision-making should consider individual factors such as treatment goals, risk tolerance and symptom severity.
